# Optimization and Modeling of *Citrobacter freundii* AD119 Growth and 1,3-Propanediol Production Using Two-Step Statistical Experimental Design and Artificial Neural Networks

**DOI:** 10.3390/s23031266

**Published:** 2023-01-22

**Authors:** Agnieszka Drożdżyńska, Jolanta Wawrzyniak, Piotr Kubiak, Martyna Przybylak, Wojciech Białas, Katarzyna Czaczyk

**Affiliations:** 1 Department of Biotechnology and Food Microbiology, Faculty of Food Science and Nutrition, Poznań University of Life Sciences, 60-624 Poznań, Poland; 2 Department of Dairy and Process Engineering, Faculty of Food Science and Nutrition, Poznań University of Life Sciences, 60-624 Poznań, Poland

**Keywords:** 1,3-propanediol, *Citrobacter freundii*, medium composition, experimental design, artificial neural networks

## Abstract

1,3-propanediol (1,3-PD) has a wide range of industrial applications. The most studied natural producers capable of fermenting glycerol to 1,3-PD belong to the genera *Klebsiella*, *Citrobacter*, and *Clostridium*. In this study, the optimization of medium composition for the biosynthesis of 1,3-PD by *Citrobacter freundii* AD119 was performed using the one-factor-at-a-time method (OFAT) and a two-step statistical experimental design. Eleven mineral components were tested for their impact on the process using the Plackett–Burman design. MgSO_4_ and CoCl_2_ were found to have the most pronounced effect. Consequently, a central composite design was used to optimize the concentration of these mineral components. Besides minerals, carbon and nitrogen sources were also optimized. Partial glycerol substitution with other carbon sources was found not to improve the bioconversion process. Moreover, although yeast extract was found to be the best nitrogen source, it was possible to replace it in part with (NH_4_)_2_SO_4_ without a negative impact on 1,3-PD production. As a part of the optimization procedure, an artificial neural network model of the growth of *C. freundii* and 1,3-PD production was developed as a predictive tool supporting the design and control of the bioprocess under study.

## 1. Introduction

1,3-propanediol (1,3-PD) has a wide range of applications in the manufacture of polymers, adhesives, cosmetics, detergents, solvents, laminates, lubricants, and medicines [1,2]. 1,3-PD can be produced by chemical synthesis or microbial fermentation. However, chemical synthesis is expensive and generates waste streams containing environmental pollutants. Thus, the research on the production of 1,3-PD is focused on microbial fermentation, which is a more environmentally favorable process and creates the opportunity to use raw glycerol as a carbon source [3,4]. Glycerol can be converted to 1,3-PD by various bacteria, including those of the genera *Klebsiella*, *Citrobacter*, and *Clostridium* [5,6,7,8]. The enterobacterial species are paid attention to because of their substrate tolerance, productivity, and high yield [2,9,10]. Reported concentrations of 1,3-PD obtained using *Citrobacter freundii* range from 4.35 g/L to 68.1 g/L and depend on the particular strain, medium composition and culture conditions [7,11].

Reports are available that focus on the optimization of parameters that affect 1,3-PD production. Among the methodologies used in such studies, both one-factor-a-time (OFAT) and multifactorial design-of-experiment (DOE) approaches can be encountered. A wide range of variables can be selected for optimization, including process conditions (temperature, pH, sparging/agitation) and the chemical composition of the medium. Proper statistical methods, such as the Plackett–Burman design, allow the determination of factors that have a significant impact on the fermentation. These parameters can then be further optimized in a more comprehensive manner with central composite, Box–Behnken, or other designs. As the composition of the medium is critical, with the major roles played by concentrations of nutritional sources [3], a large part of the research concentrates on it. Regarding the production of 1,3-PD, most of the optimization studies use either *Clostridium* [12,13] or *Klebsiella species* [14,15,16,17,18]. Thus, there is only limited information on the relationship between the medium composition and 1,3-PD production by *C. freundii*.

Apart from the optimization of process parameters, models that describe the dynamics of biological processes facilitate the quantitative analysis of alternative ways of process intensification [19]. In recent years, machine learning-based techniques have gained prominence as analytical tools for predictive modeling based on their good performance in simulating complex and non-linear phenomena [20,21,22,23]. One of the significant advantages of these techniques that include artificial neural networks (ANN) is the lack of the need to make initial assumptions, which are necessary when using kinetic equations. Modeling and simulation problems handled with the use of these techniques can be solved without any a priori knowledge on the relationships existing in the considered systems [24].

In this study, the influence of various medium components on 1,3-PD production by *C. freundii* AD119 was studied. Various minerals were screened for those with a significant effect on 1,3-PD production with the use of the Plackett–Burman design. Moreover, the optimal concentrations of selected minerals were investigated by employing response surface methodology with a central composite design. The current study also addressed the influence of carbon and nitrogen sources in qualitative and quantitative aspects. Finally, a model simulating the growth of *C. freundii* and the production of 1,3-PD was developed using artificial neural networks (ANN).

## 2. Materials and Methods

### 2.1. Microorganisms

*C. freundii* AD119 strain was isolated from pickled vegetables [25]. The strain was deposited in the Polish Collection of Microorganisms under the number B/00044.

### 2.2. Media

#### 2.2.1. Mineral Requirements

For mineral optimization studies, Plackett–Burman design was used. Each medium contained a carbon source (glycerol—50 g/L), nitrogen source ((NH_4_)_2_SO_4_—3 g/L), and phosphate buffer. Minerals were selected based on literature data. The quantities of other examined minerals in the media were dependent on the experimental design (Table 1 and Table 2).

The pH of each medium was adjusted to 7.0 and the media were autoclaved for 20 min at 121 °C. The culture was maintained at 30 °C for 168 h without stirring. Quantitative optimization of MgSO_4_ and CoCl_2_ was performed using response surface methodology. The experimental designs and responses are shown in Table 3.

The mathematical function for the response (1,3-PD concentration) is presented below (Equation (1)):

y = b_0_ + b_1_A + b_2_B + b_12_AB + b_11_A^2^ + b_22_B^2^ (1)

where y—predicted yield of the response, b_0_—intercept; b_1_, b_2_—linear coefficients; b_12_ -interaction coefficient; b_11_, b_22_—quadratic coefficients.

Validity of the model was confirmed by performing three validation experiments using factor values determined as optimal. Comparison of the actual results with the interval of confidence of the calculated response was then performed.

#### 2.2.2. Carbon Source Requirements

For optimization of carbon source, 20% of glycerol was substituted with different carbon sources such as citric acid, glucose, fructose, xylose, mannose, arabinose, galactose, sucrose, lactose, starch, maltose, and carboxymethyl cellulose. For optimization of glycerol concentration, media containing different starting glycerol concentrations (ranging from 0 to 100 g/L) were used. Both pure and biodiesel-derived waste glycerol (Archer Daniels Midland Company (ADM), Malbork, Poland) were used.

#### 2.2.3. Nitrogen Source Requirements

**Figure 1:**
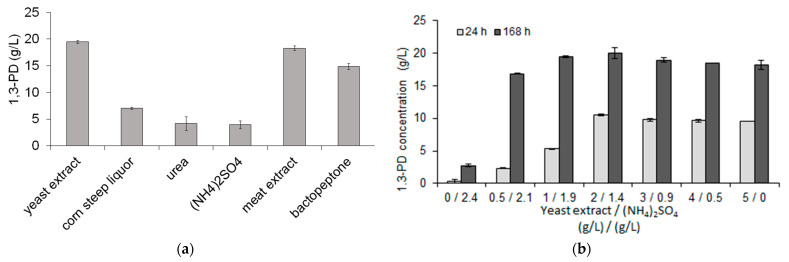
Influence of various nitrogen sources on 1,3-PD production (**a**) and effect of yeast extract replacement by (NH_4_)_2_SO_4_ on 1,3-PD production (**b**).

Different nitrogen-containing compounds (yeast extract, meat extract, bactopeptone, urea, corn steep liquor, and (NH_4_)_2_SO_4_) in the concentration of 5 g/L were used to optimize the nitrogen source in the medium. For optimization of yeast extract concentration, media containing different starting yeast extract concentrations (ranging from 0.75 to 10 g/L) were used. Moreover, partial substitution of yeast extract with (NH_4_)_2_SO_4_ was examined. Each medium contained 0.5 g/L of nitrogen originating from yeast extract and (NH_4_)_2_SO_4_ in different proportions (Figure 1b).

#### 2.2.4. Influence of Vitamin and Organic Acids

To test the influence of the addition of selected organic acids and vitamin B_12_ on 1,3-PD production, media supplemented with vitamin B_12_ (3.7 μM), fumaric acid (25 mM), and the mixture of organic acids (fumaric acid (3.8 mM), citric acid (3.8 mM), and succinic acid (3.8 mM)) were prepared.

### 2.3. Fed-Batch Experiment in Bioreactor

Fed-batch bioreactor fermentation was conducted in a 5 L bioreactor Biostat B Plus (Sartorius, Germany) with a working volume of 1 L. The optimized medium was inoculated with 10% (*v*/*v*) of inoculum. The process conditions were as follows: temperature, 30 °C; agitation, 80 rpm; pH, 7.0. Glycerol supply was coupled to the delivery of KOH—a single solution composed of 20% KOH and 41% glycerol was used for controlling pH. As the fermentation progressed, the automatic pH regulation system controlled the delivery of both the neutralizing agent and glycerol. Thus, the carbon source was simultaneously delivered at a rate synchronized with the rate of acid generation by the bacteria. 

### 2.4. Analysis of Fermentation Broth

After fermentation, samples were diluted 10-fold and filtered through a 0.45 μm syringe filter before analysis. Glycerol and 1,3-PD were quantified by HPLC using Agilent Technologies 1200 series chromatograph (Agilent Technologies, Santa Clara, CA, USA) equipped with a refractive-index detector (G1362A). Analyses were performed isocratically at 40 °C with 0.6 mL/min flow rate of 0.5mN H_2_SO_4_ on Rezex ROA-Organic Acid, 300 × 7.8 mm (Phenomenex, Torrance, CA, USA) column. Standard solutions (all from Sigma-Aldrich, Merck, Germany) were used to identify peaks in chromatograms, and peak area was used for quantification. ChemStation for LC 3D systems (Agilent Technologies, Santa Clara, CA, USA) was used to analyze chromatographic data. Optical density at 600 nm was used to analyze bacterial growth.

### 2.5. Artificial Neural Network Model Development

ANNs were used to develop a model for the evaluation of the suitability of solutions containing glycerol for *C. freundii* growth and 1,3-PD production. The experimental data collected during the fermentation processes, i.e., 1,3-PD concentration and optical density, were used to build the ANN model. Before the model design, the experimental data set (308 vectors) was divided randomly into three groups, i.e., the training data set used to build the model, the test data set used to verify the network quality during the training process, and the validation data set (not involved in the construction process) used to verify the network quality after the model elaboration. The ratio of data points in these data sets was 70:15:15, respectively.

The ANN model was built on the basis of multilayer perceptron (MLP), which has been proven in previous studies to work well in regression problems, especially in the context of microbial growth modelling [26,27,28,29]. This type of fully connected feed-forward network consisting of three layers, i.e., input, hidden, and output layers, was also used in our research. The independent variables of the fermentation process (i.e., type of glycerol source, glycerol content in the culture medium, fermentation time) were used as inputs for the model. The outputs were designated based on the dependent variables, i.e., the concentration of 1,3-PD and the optical density (a measure of *C. freundii* population growth). In the output neurons, a linear function was used as the activation (transfer) function. The development of the hidden layer structure was less straightforward, because there are no simple and generally accepted principles for its design [30,31]. There are some rules of thumb, which allow estimation of the number of neurons in the hidden layer [28,32,33]. Nevertheless, the most common approach used to determine the number of neurons in the hidden layer is the trial-and-error method, which was also used in our study. Networks with a single hidden layer containing from 3 to 12 neurons with various types of activation function in these neurons (linear (Lin), logistic (Log), exponential (Exp), and hyperbolic tangent (Tanh)) were examined. For each studied topology, 1000 neural network models were tested (40,000 networks in total). Simulations were performed using Statistica 13.3 software (StatSoft, Tulsa, OK, USA). Network training was aimed at minimizing the error between the target output vector and the ANN-calculated output signal. To this end, the supervised learning algorithm Broyden—Fletcher—Goldfarb—Shanno (BFGS) was used to fit the optimal values of the activation function coefficients, the values of inter-neural synaptic weights and biases. The prediction performance of all the tested ANN models was evaluated based on the errors computed for the learning, test, and validation data sets. Additionally, the capability of the final ANN model predicting the level of *C. freundii* population and the production of 1,3-PD was assessed on the basis of the explained variation (R^2^), mean absolute error (MAE), and the root mean square error (RMSE).

## 3. Results and Discussion

A sequential approach was applied in the present study to optimize the medium for 1,3-PD production by *C. freundii*. First, qualitative optimization of minerals, carbon, and nitrogen sources was performed. This was followed by quantitative optimization of selected variables.

### 3.1. Qualitative Optimization

#### 3.1.1. Plackett–Burman Design—Mineral Composition Optimization

The Plackett–Burman design was employed to evaluate the influence of 11 minerals on 1,3-PD production by *C. freundii* AD119. Minerals were selected based on available literature data. Experimental levels tested in this design are shown in Table 2. The data showed a wide variation of 1,3-PD production that ranged from 0 to 14.29 g/L. While relatively high 1,3-PD production was observed when MgSO_4_ was added to the medium, there was almost no 1,3-PD production when CoCl_2_ was present in a medium devoid of MgSO_4_ (8.20–14.29 g/L of 1,3-PD and 0–0.61 g/L of 1,3-PD, respectively, Table 2). To determine the significance of the impact of the variables on the response, statistical analysis of variance (ANOVA) was performed (Table 4).

**Figure 2:**
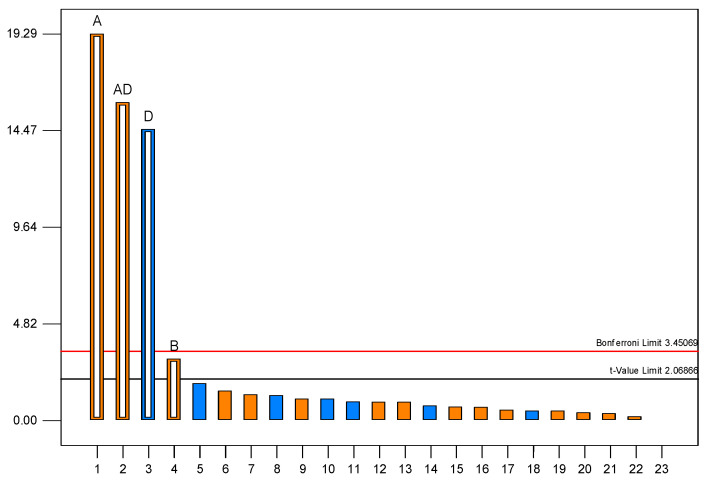
Pareto chart. A-MgSO_4_, D-CoCl_2_, B-CaCl_2_.

Three variables, namely MgSO_4_, CoCl_2_, and CaCl_2_, were found to influence the fermentation process significantly. The values of regression coefficients indicate a negative effect of CoCl_2_ and positive effects of MgSO_4_ and CaCl_2_. Furthermore, an interaction between the concentration of CoCl_2_ and MgSO_4_ was found to have a significant positive impact. The concentration of CaCl_2_ showed relatively low significance compared to the other factors and was thus not a subject of optimization in further studies (Figure 2).

There is no literature data concerning the effect of minerals (magnesium or cobalt) on 1,3-PD production by *C. freundii*. However, Huang et al. [34] showed the positive effect of cobalt ions addition on 1,3-PD production by *Klebsiella pneumoniae*. Magnesium is essential to the mechanism of cell division, and increases growth yield [35], and buffers the cell against adverse environmental effects [36]. Moreover, Kajiura et al. [37] reported the reactivation of inactivated glycerol dehydratase of *K. pneumoniae* in the presence of free adenosylcobalamin, Mg^2+^, and ATP. Cobalt ions also might play a role in the reactivation of this enzyme, as the B_12_-dependent glycerol dehydratase of *C. freundii* contains an atom of cobalt in its center [38,39]. Interestingly, the addition of cobalt ions to the medium inhibited cell growth and 1,3-PD production. Similar observations were made by Ranquet et al. [40] and Babai [41], who noticed cell growth inhibition of *Escherichia coli* in the presence of cobalt in a medium. Moreover, Babai [41] found that the toxicity of cobalt was markedly reduced in the presence of magnesium. Thus, the interaction between Mg^2+^ and Co^2+^ concentration demanded further studies. It was done using a central composite design during the quantitative optimization of selected variables.

#### 3.1.2. Carbon Source Optimization

**Figure 3:**
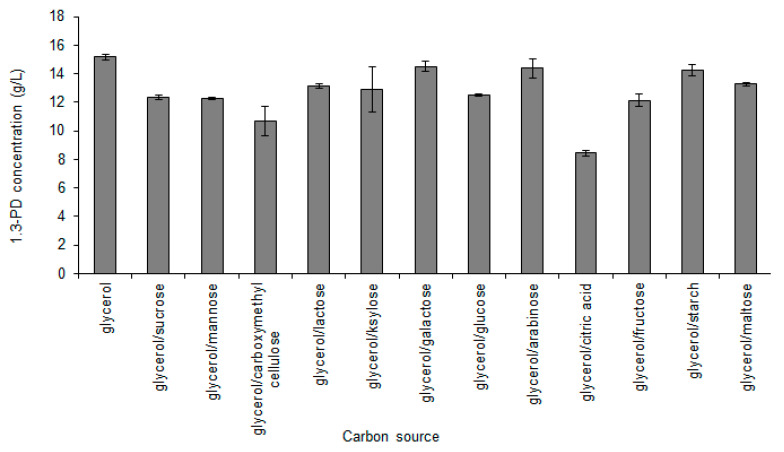
Effects of co-substrates addition on 1,3-PD production by *C. freundii* AD119.

Glycerol is the only carbon source that can be converted to 1,3-PD by non-engineered microorganisms. No microorganisms have the ability to ferment sugars directly to 1,3-PD [1]. Sugars, however, can be used as additional carbon sources. An example of such an approach can be found in the study by Abbad-Andaloussi et al. [42], where it was reported that the introduction of glucose into the medium increased the amount of glycerol metabolized through the 1,3-PD pathway. In the co-substrate–glycerol medium, the co-substrate was metabolized by the cells to produce energy, whereas glycerol was used mainly for the utilization of reducing power and the production of 1,3-PD. Thus, 20% of glycerol was replaced with other carbon sources. In almost all cases where co-substrates were used, a decrease in the production of 1,3-PD was observed, from 15.2 ± 0.2 g/L of 1,3-PD (control) to the range of 8.4 ± 0.2–13.3 ± 0.2 of 1,3-PD (Figure 3).

In some cases (galactose, arabinose, starch), the effect was negligible (14.5 ± 0.3, 14.4 ± 0.7 and 14.3 ± 0.4 g/L of 1,3-PD, respectively, compared to 15.2 ± 0.2 g/L in control). Similar results were obtained by Metsoviti et al. [43], who tested the effect of partial glycerol substitution with glucose and reported no increase in 1,3-PD production. An increase in 1,3-PD production, productivity, and yield was observed only when glucose was added to the medium with no change to the concentration of glycerol [15,44,45].

#### 3.1.3. Nitrogen Source Optimization

The impact of both organic and inorganic nitrogen sources on 1,3-PD production by *C. freundii* AD119 was investigated. In general, media that contained organic nitrogen sources were found to yield better results (Figure 1a). Organic nitrogen sources are known to contain vitamins and growth factors [46,47]. Maximum 1,3-PD production of 19.47 ± 0.3 g/L was obtained with the use of yeast extract. It was followed by a result of 16.44 ± 0.23 g/L of 1,3-PD determined in cultures that contained meat extract. Similar results were obtained by Jalasutram and Jetty [15]. They found that among 12 tested nitrogen sources, yeast extract was the optimum nitrogen source for the production of 1,3-PD by a *K. pneumoniae* strain. Yeast extract was thus selected for use in further research.

### 3.2. Quantitative Optimization

#### 3.2.1. Optimization of Nitrogen Sources

To determine the optimum amount of nitrogen source for 1,3-PD production, media containing yeast extract at concentrations ranging from 0.75 to 10 g/L were prepared. As yeast extract concentration increased from 0.75 to 5 g/L, 1,3-PD, production also increased (Table 5).

Increasing the yeast extract concentration further did not result in higher 1,3-PD production. Maximum production was observed at 5 g/L of yeast extract. In further studies, aimed at reduction of the content of this expensive component of the medium, the possibility of partial replacement of yeast extract by (NH_4_)_2_SO_4_ was investigated. The results showed that yeast extract had a positive effect on the formation of 1,3-PD by *C. freundii* AD119 (Figure 1b). The reduction of yeast extract to 0.5 g/L caused an almost five-fold reduction of 1,3-PD concentration (from 9.6 ± 0.1 to 2.3 ± 0.1 g/L) obtained after 24 h of fermentation. The addition of 2 g/L of yeast extract and 1.4 g/L of (NH_4_)_2_SO_4_ to the medium allowed obtaining the maximum 1,3-PD concentration after 24 h of fermentation. Further studies showed that reduction of (NH_4_)_2_SO_4_ to the level of 1.1 g/L did not decrease 1,3-PD production (data not shown), thus the mixture of 2 g/L of yeast extract and 1.1 g/L of (NH_4_)_2_SO_4_ was used for further studies. Other scientists also tried to avoid the usage of yeast extract in media. Himmi et al. [48] replaced all yeast extract with biotin. *Clostridium butyricum* was able to produce the same amount of 1,3-PD in the medium without yeast extract, but the process was prolonged. Pflugl et al. [49] replaced yeast extract with vitamin B_12_, riboflavin, and nicotinic acid, and obtained a 1,3-PD concentration comparable to the cultivation with yeast extract. Their medium, however, contained other organic nitrogen sources (meat extract and casein peptone) besides yeast extract. Dietz and Zeng [50] used yeast extract-free medium containing, among others, citric acid, L-cysteine, biotin, and pantothenate. Results showed by these scientists are promising, as no prolongation of the fermentation was observed, and a high concentration of 1,3-PD was obtained. However, L-cysteine, biotin, and pantothenate are expensive components.

#### 3.2.2. Carbon Sources Optimization

**Figure 4:**
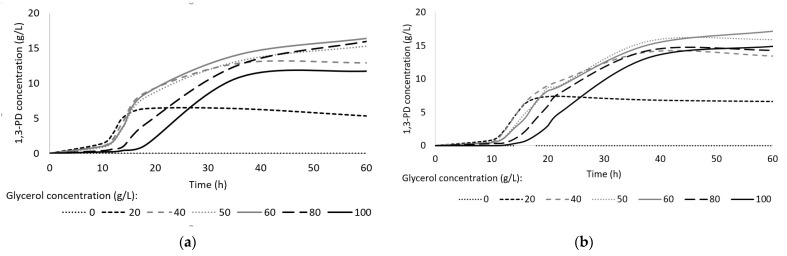
Effect of initial glycerol concentration on 1,3-PD production using pure glycerol (**a**) and crude glycerol (**b**).

To determine the optimum glycerol concentration for 1,3-PD production, media containing from 0 to 100 g/L of glycerol were prepared. Both pure glycerol and crude glycerol from biodiesel production were used, and the results were similar for both these substrate types. As glycerol concentration increased, the lag phase was prolonged and the rate of 1,3-PD production decreased (Figure 4).

**Figure 5:**
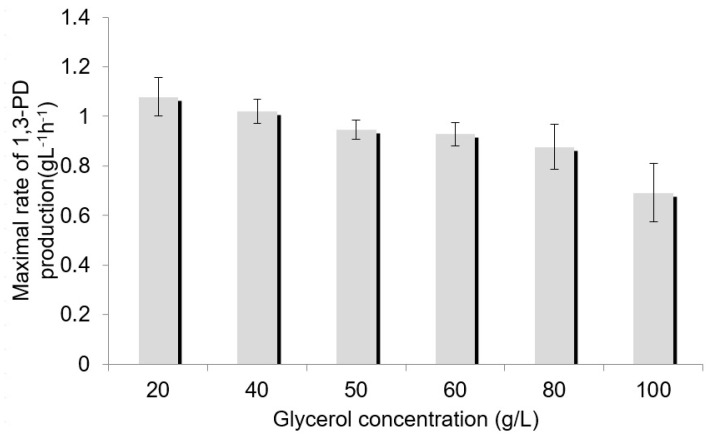
Effect of initial crude glycerol concentration on the maximum rate of 1,3-PD production.

The maximal rate of 1,3-PD production reached the highest value for 20 and 40 g/L glycerol in the medium (Figure 5). Thus, the concentration of glycerol chosen for use in further studies was 40 g/L. It is known that excessive glycerol concentration (70–90 g/L) can result in a decrease in both productivity and yield [15,51]. The optimum glycerol concentration in the medium for bacteria belonging to the Enterobacteriaceae family was found to be between 20 and 60 g/L [7,52,53].

#### 3.2.3. Minerals Optimization—Central Composite Design

Based on the results of the initial screening with Plackett-Burman design, a central composite design was developed for variables that were found to affect 1,3-PD production significantly. The design matrix and the values of 1,3-PD concentration after 24 h of fermentation (response) are given in Table 3. There was no 1,3-PD production in run no. 2, where no magnesium sulfate was added and the highest concentration of cobalt chloride was used. The highest 1,3-PD production was observed in media containing both magnesium sulfate and cobalt chloride. This confirmed the positive interaction and influence of the tested compounds on 1,3-PD production. Statistical analysis of variance (ANOVA) was performed (Table 6) and the following Equation (2) was formulated:

(1,3-PD concentration) = 5.11439 − 0.016803 A + 20.20390 B + 0.54462 AB − 0.011040 A^2^ − 23.77220 B^2^ (2)

**Figure 6:**
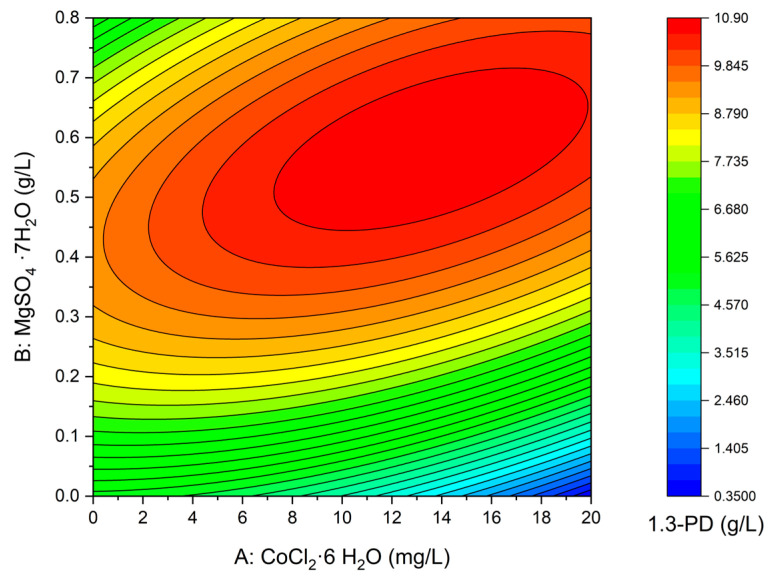
Response surface for the 1,3-PD production according to the central composite design.

The factors A and B are specified in their real units. The model showed significant positive linear effects for magnesium. An interaction between magnesium and cobalt was found to have a positive effect of high significance, which indicates that both factors are required for effective 1,3-PD production. The coefficients of quadratic variables, i.e., b_11_ and b_22_ in Equation (2), had both negative signs, indicating the existence of a maximum point in the model. In Figure 6, the calculated response surface is shown. The analysis of variance demonstrated that the presented model is statistically significant (*p* < 0.0001). The R^2^ value of 0.99 indicated that only 1% of the total variation is not explained by the model. The adjusted R^2^ value of 0.98 is also satisfactory and confirms the significance of the model.

Equation (2) was used to predict the maximum value of 1,3-PD production, and thus validate the model. The prognosed response was 10.86 g/L of 1,3-PD (confidence interval from 10.44 to 11.28 g/L) with CoCl2·6 H_2_O and MgSO4 7H_2_O concentrations at 13.55 mg/L and 0.58 g/L, respectively. All other factors (concentrations of glycerol, yeast extract, (NH4)_2_SO_4_) were kept at their optimal levels determined in the previous stages of the study. In three validating experiments, 1,3-PD concentrations of 10.86, 11.0, and 10.7 g/L were obtained, confirming the model validity.

#### 3.2.4. Addition of Organic Acids and Vitamin B12 to the Culture Medium

**Figure 7:**
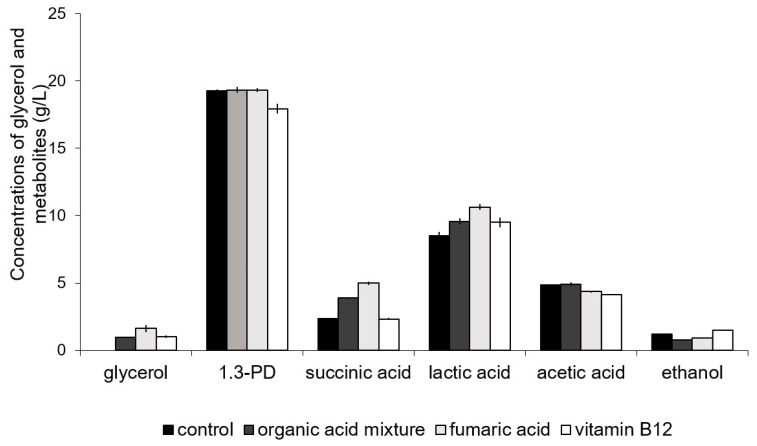
Effect of the addition of organic acids and vitamin B_12_ on metabolite production and glycerol utilization by *C. freundii* AD119.

It is reported that the addition of organic acids (e.g., succinic acid, citric acid fumaric acid) and vitamin B_12_ may benefit 1,3-PD production by *Klebsiella oxytoca* or *K. pneumoniae* [34,54,55]. However, there is no information about the influence of the above-mentioned substances on 1,3-PD production by *C. freundii*. For this reason, experiments with the addition of vitamin B_12_ (5 mg/L), fumaric acid (25 mM), and the organic acid mixture (fumaric acid (3.8 mM), citric acid (3.8 mM), and succinic acid (3.8 mM)) to the fermentation broth were performed. Obtained results are presented in Figure 7.

No significant increase in 1,3-PD production was observed in the cultures with organic acids. The addition of vitamin B_12_ caused a significant decrease in 1,3-PD production (from 19.25 ± 0.13 to 17.93 ± 0.37 g/L). These results are similar to the ones reported by Jalasutram and Jetty [15] and Jun et al. [56]. The lack of a positive effect of vitamin B_12_ on the process might have been a result of the ability of the strain to synthesize this vitamin or the fact that the glycerol dehydratase of this bacterial strain is B_12_-independent [15]. A sufficient supply of vitamin B_12_ with the yeast extract contained in the medium is another possibility [56].

#### 3.2.5. Fed-Batch Bioreactor Experiment

**Figure 8:**
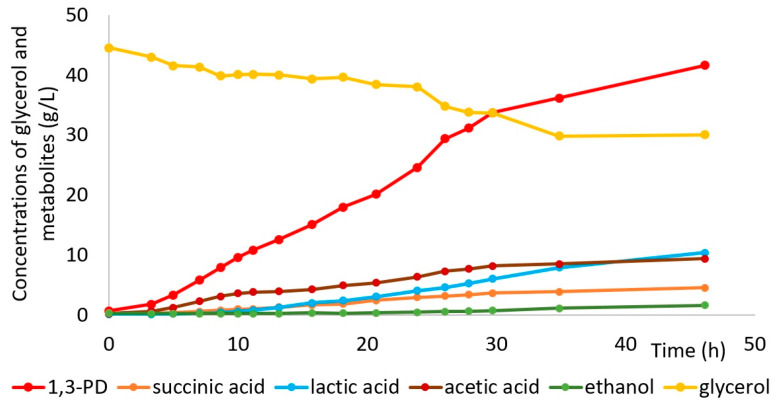
Concentration changes of glycerol and metabolites with respect to fermentation time.

Following the complete optimization process, a fed-batch culture with *C. freundii* AD119 was performed under the optimized conditions which yielded 41.7 g/L of 1,3-PD in a 48-h fermentation (Figure 8). The obtained 1,3-PD concentration was relatively high, which was the result of the feeding strategy. The glycerol supply rate was coupled to the rate at which KOH was delivered since both substances were present in the solution used for pH regulation. Acids constitute the main by-products of 1,3-PD fermentation, and their level reflects the amount of substrate consumed. With the rate of glycerol supply coupled with the rate at which the main by-products (acids) are formed, the system self-adapts to the kinetics of bacterial metabolism. Such an approach prevents sudden variations in substrate concentration over the course of the process (Figure 8). As evidenced by the results, the ratio of KOH and glycerol amounts demands further optimization in order to enable complete substrate utilization.

Lactic and acetic acids were the main by-products (both about 10 g/L). Ethanol and succinic acid were also present in the fermentation broth, but at a lower level (both below 1 g/L). Qualitatively, such a profile of by-products is characteristic of *C. freundii* [7,11,57].

The ability to ferment glycerol to 1,3-PD is a known feat among *C. freundii* strains. The actual efficiency of this process is, however, dependent on the capacity of the strain itself and the process conditions, which include medium composition. Over the years, many studies of this bioconversion process have been performed in batch, fed-batch, immobilized, and continuous cultures (Table 7). A fed-batch fermentation using *C. freundii* FMCC-B 294 (VK-19) that yielded 68.1 g/L 1,3-PD was reported, proving the potential of this species. Our result, 41.7 g/L of 1,3-PD in fed-batch culture, indicates that further studies, including the optimization of culture conditions, may lead to improved production of 1,3-PD by *C. freundii* AD119.

### 3.3. Artificial Neural Network Model

Modeling biological processes has always been a challenge for scientists, as simulating dynamic and continuous processes with a single mathematical equation that must fulfill certain assumptions is not an easy task. The use of intelligent simulation in the design and optimization of biological processes offers new possibilities related to the fast prediction of microbial activity through computer processing, without or before microbial detection [65]. Artificial neural networks are one of the machine-learning techniques used in such applications. They enabled the prediction of the population dynamics of *Pseudomonas aeruginosa* in Frankfurter sausage containing *Satureja bachtiarica* extracts [26]. Ebrahimi et al. [27] compared ANN and multivariate regression (MLR) models for the prediction of *Azotobacteria* population in soil under different land uses. The performance of ANN was found to be better than MLR. In another study, ANNs and kinetic equations were used to model and simulate *Streptomyces peucetius* var. *caesius* N47 cultivation and ε-rhodomycinone production, and ANNs were found to be the superior approach [24].

**Figure 9:**
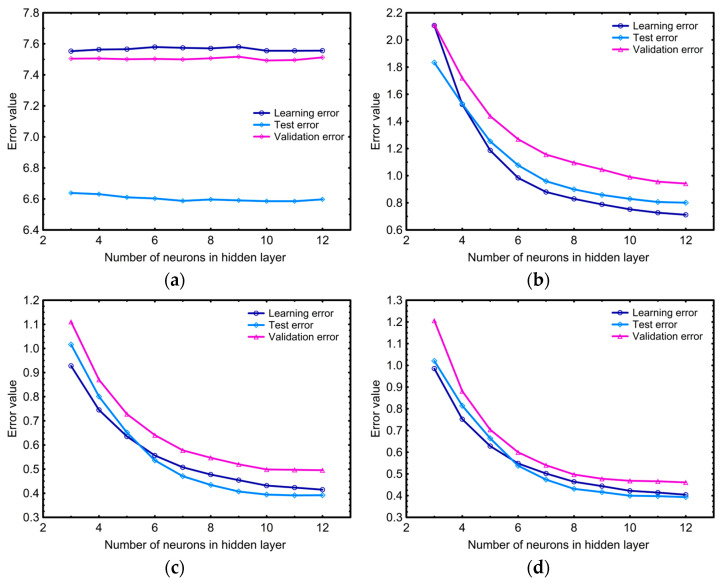
Changes in mean values of training, test, and validation errors of MLP networks applied to modelling of *C. freundii* population growth and 1,3-PD production depending on the number of neurons in the hidden layer equipped with an activation function in form of (**a**) a linear (Lin), (**b**) an exponential (Exp), (**c**) a logistic function (Log), and (**d**) a hyperbolic tangent (Tanh).

In this study, ANNs were used to develop a model for predicting the growth of *C. freundii* population and 1,3-PD production as a function of the type of glycerol source, glycerol concentration in the culture medium, and incubation time (ANN-M_Cf_PD_). Determination of a neural network model is a process that requires experience. The process of ANN structure development includes the selection of an appropriate structure of the hidden layer in the network topology, the definition of a transfer function in its neurons, and the determination of both values of the transfer function coefficient and the values of synaptic weights and biases (ANN model parameters). A total of 40,000 networks were tested during the model design process. They differed in the number of neurons in the hidden layer and the activation functions operating in them. The predictive quality of the constructed networks was assessed on the basis of the values of learning, test, and validation errors. Changes in the average value (out of 1000) of the mentioned network errors are shown in Figure 9 as a function of the number of neurons in the hidden layer and with respect to the type of their activation function.

The presented graphs depict that the networks with the Lin activation function were characterized by the highest and constant errors regardless of the network topology. These outcomes suggested that mentioned structures are not suitable to simulate the growth of *C. freundii* population and the production of 1,3-PD. Significantly better results were obtained for networks containing non-linear activation functions (Log, Tanh, and Exp) in the neurons of the hidden layer. The common feature of these three groups of networks was the initial decrease in the value of all errors, and then their stabilization at an almost constant level for networks containing eight and more neurons in the hidden layer. The final values of computed errors for the networks, which contained Log and Tanh activation functions in the neurons of the hidden layer, were at comparable levels. In the case of the networks with the Exp transfer function, the values of the analyzed errors in hidden neurons were slightly higher. When developing a neural network, it is important to remember that to ensure accurate simulations and prediction of the ANN model, the network topology cannot be too small, as it may constitute a powerless structure for a given modeling problem. On the other hand, too excessive network topology usually leads to “memorization” of individual cases and poor ability to generalize when working on new data [66]. Taking into account the above considerations, further research on designing the structure of the ANN model for the development of the *C. freundii* population and the production of 1,3-PD was carried out on the basis of the networks, in which the hidden layer consisted of no more than eight neurons. The network with the best predictive quality, expressed as the lowest sum of learning, test, and validation errors, was adopted as the ANN-M_Cf_PD_. The metrices of this MLP network model are depicted in Table 8. The selected network contained eight neurons in the hidden layer equipped with Tanh as a transfer function, and it constituted a compromise between simplicity of structure and generalization capability.

**Figure 10:**
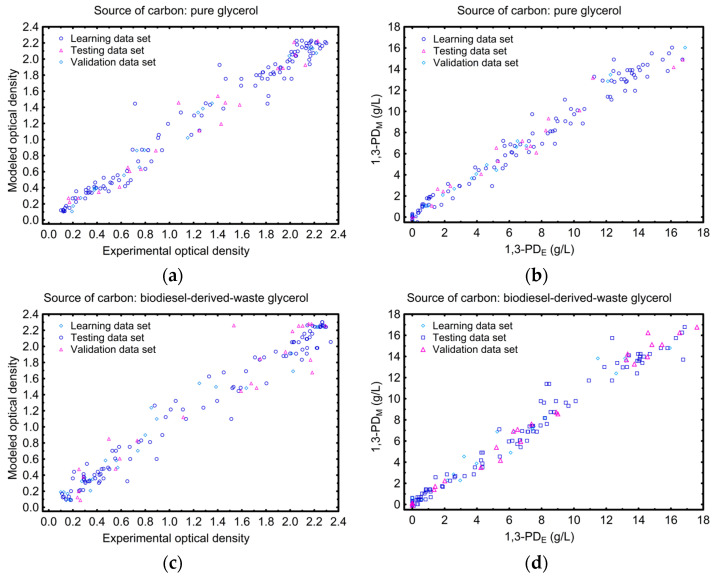
A comparison of the artificial neural network model (ANN-M_Cf_PD_) predictions against the experimental data describing the growth of *C. freundii* population and the production of 1,3-propanediol on pure (**a**,**b**) and biodiesel-derived waste glycerol (**c**,**d**), respectively.

The predictive effectiveness and the possibility of practical application of the selected ANN model were assessed using statistical indicators computed for all data sets (training, test, and validation). Figure 10 shows good agreement between the model predictions and the experimental points, which was confirmed by the high values of R^2^ (Table 9). Additionally, low values of both MAE and RMSE for training, test, and validation data sets showed the high-performance accuracy of the model. It is worth noting that an extremely important aspect related to the application of the model is its performance when operating on new process data, unrelated to the model construction. Hence, the values of the used indicators, determined on the basis of validation data set, confirmed not only the high precision of the prediction, but also revealed the high generalization ability of the developed ANN model.

The obtained results suggest that the elaborated ANN-M_Cf_PD_ model can be a useful tool for simulating the process of fermentation of glycerol-based substrates by *C. freundii*. It could be applied for the determination of the suitability of wastes containing this compound for the production of 1,3-propanediol.

## 4. Conclusions

In the presented study, medium components that play significant roles in shaping the efficiency of glycerol conversion to 1,3-PD in *C. freundii* AD119 were identified and optimized with the use of one-factor-at-a-time (OFAT) and two-step statistical experimental design (Plackett–Burman and central composite design [CCD]) approaches. Among the studied mineral components, MgSO_4_ and CoCl_2_ were found to have the greatest impact. Their respective optimal concentrations, found using CCD, were 13.55 mg/L (CoCl_2_·6H_2_O) and 0.58 g/L (MgSO_4_·7H_2_O). Yeast extract has shown to be the best nitrogen source. It was, however, possible to partially replace it with (NH_4_)_2_SO_4_. A partial substitution of glycerol with other carbon sources resulted in decreased process effectiveness. Moreover, the developed ANN-M_Cf_PD_ model was able to predict the growth of *C. freundii* and 1,3-PD production in batch cultivation in media containing both pure and crude glycerol in a wide range of concentrations. The obtained results prove that ANN models have the potential to become a useful decision-making tool for supporting the design and control of the 1,3-PD production bioprocess. This research fills an important gap, as no comprehensive reports exist on the nutrient and mineral requirements for 1,3-PD production by *C. freundii*. It also indicates that with further optimization, the bioconversion of glycerol to 1,3-PD by *C. freundii* AD119 can be effectively improved. With an optimized medium, it seems natural that other culture conditions should also be taken into account in the future.

## Figures and Tables

**Table 1:** Experimental ranges and levels of the selected minerals tested via the Plackett–Burman design.

Factor	Symbol	Ranges and Levels		
		1	0	−1
MgSO_4_∙7H_2_O (g/L)	A	0	0.2	0
CaCl_2_ (g/L)	B	0	0.05	0.1
FeSO_4_∙7H_2_O (mg/L)	C	0	5	10
CoCl_2_ 6H_2_O (mg/L)	D	0	5	10
MnSO_4_∙H_2_O (mg/L)	E	0	8.5	17
ZnCl_2_ (mg/L)	F	0	1	2
H_3_BO_3_ (mg/L)	G	0	0.025	0.05
Na_2_MoO_4_∙2H_2_O (mg/L)	H	0	0.02	0.04
NiCl_2_∙6H_2_O (mg/L)	J	0	0.01	0.02
CuCl_2_∙2H_2_O (mg/L)	K	0	0.05	0.1
NaCl (g/L)	L	0	0.25	0.5

**Table 2:** Plackett–Burman design for 11 variables with coded values along with observed results for 1,3-PD production.

No.	A	B	C	D	E	F	G	H	J	K	L	1,3-PD Production
1	1	1	−1	1	1	1	−1	−1	−1	1	−1	12.71
2	−1	1	1	−1	1	1	1	−1	−1	−1	1	11.41
3	1	−1	1	1	−1	1	1	1	−1	−1	−1	11.76
4	−1	1	−1	1	1	−1	1	1	1	−1	−1	0.05
5	−1	−1	1	−1	1	1	−1	1	1	1	−1	9.76
6	−1	−1	−1	1	−1	1	1	−1	1	1	1	0.02
7	1	−1	−1	−1	1	−1	1	1	−1	1	1	12.07
8	1	1	−1	−1	−1	1	−1	1	1	−1	1	14.10
9	1	1	1	−1	−1	−1	1	−1	1	1	−1	14.29
10	−1	1	1	1	−1	−1	−1	1	−1	1	1	0.61
11	1	−1	1	1	1	−1	−1	−1	1	−1	1	9.03
12	−1	−1	−1	−1	−1	−1	−1	−1	−1	−1	−1	9.34
13	0	0	0	0	0	0	0	0	0	0	0	11.54
14	0	0	0	0	0	0	0	0	0	0	0	11.48
15	0	0	0	0	0	0	0	0	0	0	0	11.32
16	−1	−1	1	−1	−1	−1	1	1	1	−1	1	8.03
17	1	−1	−1	1	−1	−1	−1	1	1	1	−1	13.55
18	−1	1	−1	−1	1	−1	−1	−1	1	1	1	8.86
19	1	−1	1	−1	−1	1	−1	−1	−1	1	1	8.20
20	1	1	−1	1	−1	−1	1	−1	−1	−1	1	11.49
21	1	1	1	−1	1	−1	−1	1	−1	−1	−1	10.96
22	−1	1	1	1	−1	1	−1	−1	1	−1	−1	0.09
23	−1	−1	1	1	1	−1	1	−1	−1	1	−1	0.00
24	−1	−1	−1	1	1	1	−1	1	−1	−1	1	0.00
25	1	−1	−1	−1	1	1	1	−1	1	−1	−1	7.35
26	−1	1	−1	−1	−1	1	1	1	−1	1	−1	7.04
27	1	1	1	1	1	1	1	1	1	1	1	12.60
28	0	0	0	0	0	0	0	0	0	0	0	11.94
29	0	0	0	0	0	0	0	0	0	0	0	10.56
30	0	0	0	0	0	0	0	0	0	0	0	9.94

**Table 3:** Experimental design and experimental results for the central composite design.

No.	Design Matrix		Experimental Responses 1,3-PD (g/L)
	A: CoCl_2_·6 H_2_O (mg/L)	B: MgSO_4_·7H_2_O (g/L)	
1	0	0	5.00
2	20	0	0.00
3	0	0.8	6.05
4	20	0.8	9.77
5	0	0.4	9.52
6	20	0.4	9.62
7	10	0	4.32
8	10	0.8	9.42
9	10	0.4	9.78
10	10	0.4	10.39
11	10	0.4	10.17
12	10	0.4	10.18
13	10	0.4	10.23

**Table 4:** Statistical analysis results according to ANOVA for Plackett–Burman design.

Source	Coefficients of Regression Equation	SS	df	MS	*F*-Value	*p*-Value
Model	-	41.91	4	10.48	211	<0.0001
Intercept	2.50	-	-	-	-	-
A—MgSO_4_	0.88	18.47	1	18.47	372	<0.0001
B—CaCl_2_	0.14	0.47	1	0.47	9.46	0.0054
D—CoCl_2_	−0.66	10.48	1	10.48	211	<0.0001
AD	0.72	12.49	1	12.49	251.4	<0.0001
Residual	-	1.142	23	0.05	-	-
Lack of fit	-	1.094	19	0.06	4.77	0.0702

R^2^ = 0.97, Adj-R^2^ = 0.97, SS—sum of squares, df—degree of freedom, MS—mean square.

**Table 5:** Effect of yeast extract concentration on 1,3-PD production *C. freundii* AD119.

Yeast Extract Concentration g/L	1,3-PD g/L	Glycerol Utilization (%)
0.75	6.52 ± 0.27	24.37 ± 2.88
2	17.57 ± 0.15	73.73 ± 0.71
5	19.47 ± 0.3	84.66 ± 0.37
7.5	18.73 ± 0.26	84.76 ± 1.12
10	19.15 ± 0.06	86.09 ± 0.53

**Table 6:** Statistical analysis results according to ANOVA for the central composite design.

Source	Coefficients of Regression Equation	SS	df	MS	*F*-Value	*p*-Value
model	-	122.46	5	24.49	139.02	<0.0001
Intercept	5.11439	-	-	-	-	-
A—CoCl_2_ ·6 H_2_O	−0.016803	0.23	1	0.23	1.33	0.2869
B—MgSO_4_ ·7H_2_O	20.20390	42.23	1	42.23	239.7	<0.0001
AB	0.54462	18.98	1	18.98	107.75	<0.0001
A^2^	−0.011040	3.37	1	3.37	19.11	0.0033
B^2^	−23.77220	39.96	1	39.96	226.81	<0.0001
Residual	-	1.23	7	0.18	-	-
Lack of fit	-	1.03	3	0.34	6.81	0.0474

R^2^ = 0.99; Adj-R^2^ = 0.98; SS—sum of squares, df—degree of freedom, MS—mean square.

**Table 7:** 1,3-PD production by *C. freundii* dependent on types of culture and strain.

No.	Type of Culture	Strain	Concentration of 1,3-PD (g/L) ^1^	Ref.
1	Batch	*C. freundii* ATCC 8090	4.35	[11]
2	Batch (flask)	*C. freundii* AD119	0–8.0 ^2^	[25]
3	Batch	*C. freundii* FMCC-B 294 (VK-19)	10.1	[58]
4	Immobilized	*C. freundii*	11.3	[59]
5	Fed-batch	*C. freundii* CF-5	11.8	[60]
6	Batch	*C. freundii* Zu	12	[61]
7	Batch	*C. freundii* K2	12.4	[61]
8	Batch: Membrane bioreactor	*C. freundii*	12.4	[62]
9	Immobilized cell reactor	*C. freundii* DSM 30040	16.4	[63]
10	Immobilized	*C. freundii*	18.2	[59]
11	Batch (bioreactor)	*C. freundii* AD119	23.3	[25]
12	Batch	*C. freundii*	1.4–25.6 ^3^	[57]
13	2-stage continuous	*C. freundii* DSM 30040	41.5	[64]
14	Fed-batch	*C. freundii* AD119	41.7	This work
15	Batch	*C. freundii* FMCC-B 294 (VK-19)	45.9	[7]
16	Fed-batch	*C. freundii* VK-19 FMCC-B 294 (VK-19)	47.4	[4]
17	Fed-batch	*C. freundii* FMCC-B 294 (VK-19)	68.1	[7]

^1^ final (maximal) concentration of 1,3-PD was converted into unit g/L; ^2^ depend on the medium used; ^3^ depend on the type of glycerol used and its pretreatment.

**Table 8:** Basic information on the structure and values of learning, test, and validation error computed for MLP neural networks adopted as the neural network model to predict the *C. freundii* population and the production of 1,3-propanediol on glycerol-based media.

Network Parameters	Artificial Neural Network
	MLP 3-8-2
Number of observation points (total)	308
Learning	216
Test	46
Validation	46
Activation functions in hidden layer	Tanh
Activation functions in output layer	Lin
Learning error	0.350
Test error	0.233
Validation error	0.338
Learning accuracy	0.987
Test accuracy	0.990
Validation accuracy	0.977

**Table 9:** Values of indicators used to evaluate performance of the ANN-M_Cf_PD_ model to predict the growth of *C. freundii* population and the production of 1,3-propanediol on used glycerol-based media computerized for all data sets: L—learning, T—test, V—validation, F—full.

Statistical Index	Data Set			
	L	T	V	F
*C. freundii* population level				
Coefficient of determination (R^2^)	0.971	0.976	0.931	0.967
Root mean square error (RMSE)	0.018	0.120	0.198	0.143
Mean absolute error (MAE)	0.091	0.088	0.141	0.098
1,3-propanediol concentration				
Coefficient of determination (R^2^)	0.976	0.982	0.979	0.978
Root mean square error (RMSE)	0.826	0.672	0.798	0.801
Mean absolute error (MAE)	0.570	0.475	0.589	0.559

## Data Availability

Not applicable.
